# Chronic Discharges from the Ear and Their Treatment

**Published:** 1898-12-10

**Authors:** Macleod Yearsley

**Affiliations:** Assistant-Surgeon to the Royal Ear Hospital; Surgeon-in-Charge of the Department for Diseases of the Throat, Nose, and Ear, the Farringdon General Dispensary; Hon. Surgeon for Diseases of the Throat and Ear, the Governesses's Home, &c.


### Hospital Clinics and Medical Progress.

### CHRONIC DISCHARGES FROM THE EAR AND THEIR TREATMENT.

By Macleod Yearsley, F.R.C.S., Assistant-Surgeon to the Royal Ear Hospital; Surgeon-in-Charge of the Department for Diseases of the Throat, Nose, and Ear, the Farringdon General Dispensary; Hon. Surgeon for Diseases of the Throat and Ear, the Governesses's Home, &c.

(*Read before the South-West London Medical Society, November 9th*, 1898.)

(*Continued from page* 160.)

As regards the prognosis of middle-ear suppurations it is, generally speaking, uncertain. When it follows scarlatina, measles, influenza, or typhus, or is due to tubercle, syphilis, or other cachexias, the prognosis is less favourable than when the cause is more easily removable. When the perforation is large or in Shrapnell's membrane, or there are extensive granulations, or caries of the temporal bone, tight stricture of the Eustachian tube, formation of cholesteatomata, involvement of the facial nerve, or involvement of the mastoid antrum the prognosis is distinctly unfavourable.

We now come to the all-important subject of treatment. This will be greatly influenced by two conditions, the local changes which have been observed in the ear and the cause of the disease. Let us take the latter first. A careful examination of throat, nose, and naso-pharynx should be made in every case, and any existing condition which can act as a primary cause or tends to keep up the middle-ear trouble should be remedied. Hypertrophied tonsils and post-nasal adenoids should be removed, and it may be said here that the removal of post-nasal growth in adults is even more important than in children. One often finds that a chronic middle-ear suppuration in an adult will resist treatment until a comparatively small pad of adenoids has been removed. The operation for post-nasal growths is too well known to need description here. Personally, in common with my colleagues at the Royal Ear Hospital, I prefer to operate under general anæsthesia with Gottstein's curette or a modification of that instrument. The necessity of general treatment, more especially where there is any constitutional dyscrasia such as syphilis, anæmia, and the like, need not be insisted upon here.

The local treatment to be adopted must be, as I have said, influenced by the local condition. The chief local causes which lead to the chronicity of the suppuration are the presence of granulations and polypi, the retention and caseation of purulent material in the recesses of the tympanum, caries of the ossicles or temporal bone, the formation of cholesteatomata, and chronic inflammatory conditions of the nose and naso-pharynx. The first duty in local treatment is, then, to gain as accurate a knowledge as possible of the local condition, the second is to effect a thorough cleansing of the ear. In order to carry out the first, one must be prepared to at once remove any polypi or granulations that may be present—a simple operation, and one which in the majority of cases can be done under cocain or eucain with a small snare and the curette. The ear should be thoroughly well antisepticised first, and all superfluity of pus or *débris* removed; and when the granulations have been thoroughly got rid of the ear should be syringed with hot antiseptic, and then dressed by packing with a strip of double cyanide gauze soaked in 1 in 40 carbolic, further treatment being postponed for 24 hours. When the anatomical form of the tympanic cavity is considered, the necessity for a thorough cleansing to prevent stagnation of discharge in one or more of the many recesses is at once evident. In simple, uncomplicated cases thorough cleansing with aseptic or antiseptic fluid will often suffice to bring about a cure. This cleansing can be effected by syringing, combined with the use of the Politzer's air douche or the Eustachian catheter. When syringing the ear for the first time it is advisable to use a very gentle stream of comfortably warm fluid to guard against causing vertigo, since dizziness of sufficient severity to cause the patient to fall may occur, owing to the sudden forcible impact of the fluid through the perforation on to the fenestræ in the tympanic wall. Such vertigo, should it occur, can, however, be remedied by using the Politzer's bag or by rarefying the air in the meatus by means of Siegle's speculum. It is here necessary to give a word of warning with regard to the use of the air douche in treating chronic suppuration of the middle ear; care should be taken to employ but the gentlest force, as by using it too powerfully pus may be blown into the mastoid antrum, and the case rendered more grave by the infection of that region. This is especially the case when the suppuration affects the attic. Some otologists avoid the use of the air douche in all chronic suppurations, but if care be taken, and the douche be not used if the outlet for pus into the meatus is an easily-blocked one, no harm need result. In doubtful cases, I personally prefer to exhaust the air in the meatus by means of a Siegle's speculum. The fluids which may be used in treatment by syringing are many. Plain boiled water will sometimes suffice, or water rendered alkaline by the addition of some soda salt, the latter being often useful at the commencement to dissolve out any albuminous flakes of inspissated pus which may be retained. Usually, however, it is better to use an antiseptic, such as carbolic (1 in 40), lysol (1 to 2 per cent.), resorcin (2 to 3 per cent.), boric acid (ʒi. to a pint), perchloride of mercury (1 in 3,000 to 4,000), or permanganate of potassium. The number of times in the 24 hours that the ear should be syringed is best determined by the amount of the discharge. If the latter be profuse, the injection should be used three or four times; if only moderate, twice or sometimes even once will suffice. And it may here be said, once for all, that it is useless for a patient to attempt to syringe his own ear; it must be done for him, and in order to ensure the proper carrying out of the treatment personal instruction should be given to whatever relative or friend is willing to undertake it. Although simple syringing may cure a number of cases, one generally has to employ some other means in conjunction therewith. It must be remembered that simple antiseptic cleansing will not always suffice, because the inflammatory infiltration of the tympanic lining membrane generally requires other means for its removal.

(*To be concluded.*)

